# Impact of Pre-Analytical Factors on MSI Test Accuracy in Mucinous Colorectal Adenocarcinoma: A Multi-Assay Concordance Study

**DOI:** 10.3390/cells9092019

**Published:** 2020-09-02

**Authors:** Umberto Malapelle, Paola Parente, Francesco Pepe, Caterina De Luca, Pellegrino Cerino, Claudia Covelli, Mariangela Balestrieri, Gianluca Russo, Antonio Bonfitto, Pasquale Pisapia, Fabiola Fiordelisi, Maria D’Armiento, Dario Bruzzese, Fotios Loupakis, Filippo Pietrantonio, Maria Triassi, Matteo Fassan, Giancarlo Troncone, Paolo Graziano

**Affiliations:** 1 Department of Public Health, University of Naples Federico II, 80131 Naples, Italy; umberto.malapelle@unina.it (U.M.); francesco.pepe4@unina.it (F.P.); caterina.deluca@unina.it (C.D.L.); strategia@izsmportici.it (P.C.); gianlucar93@libero.it (G.R.); pasquale.pisapia@unina.it (P.P.); maria.darmiento@unina.it (M.D.); dario.bruzzese@unina.it (D.B.); triassi@unina.it (M.T.); 2 Unit of Pathology, Fondazione IRCCS Casa Sollievo Della Sofferenza, San Giovanni Rotondo, 71013 Foggia, Italy; paolaparente77@gmail.com (P.P.); cla.covelli85@gmail.com (C.C.); antonio.bonfitto@operapadrepio.it (A.B.); fabiolafiordelisi@gmail.com (F.F.); p.graziano@operapadrepio.it (P.G.); 3 Surgical Pathology Unit, Department of Medicine (DIMED), University of Padua, 35128 Padua, Italy; mariangela.balistreri@aopd.veneto.it (M.B.); matteo.fassan@unipd.it (M.F.); 4 Department of Clinical and Experimental Oncology, Medical Oncology Unit 1, Istituto Oncologico Veneto (IRCSS), 35128 Padua, Italy; fotios.loupakis@iov.veneto.it; 5 Medical Oncology Department, Fondazione IRCCS Istituto Nazionale Dei Tumori, 20133 Milano, Italy; filippo.pietrantonio@unimi.it; 6 Oncology and Hemato-Oncology Department, University of Milan, 20133 Milan, Italy

**Keywords:** predictive molecular pathology, IHC, fully automated RT—PCR, microfluidic, mCRC

## Abstract

Immunohistochemistry (IHC) and polymerase chain reaction (PCR) and fragment separation by capillary electrophoresis represent the current clinical laboratory standard for the evaluation of microsatellite instability (MSI) status. The importance of reporting MSI status in colorectal cancer is based on its potential for guiding treatment and as a prognostic indicator. It is also used to identify patients for Lynch syndrome testing. Our aim was to evaluate pre-analytical factors, such as age of formalin-fixed and paraffin-embedded (FFPE) block, neoplastic cell percentage, mucinous component, and DNA integrity, that may influence the accuracy of MSI testing and assess the concordance between three different MSI evaluation approaches. We selected the mucinous colorectal cancer (CRC) histotype for this study as it may possibly represent an intrinsic diagnostic issue due to its low tumor cellularity. Seventy-five cases of mucinous CRC and corresponding normal colon tissue samples were retrospectively selected. MMR proteins were evaluated by IHC. After DNA quality and quantity evaluation, the Idylla™ and TapeStation 4200 platforms were adopted for the evaluation of MSI status. Seventy-three (97.3%) cases were successfully analyzed by the three methodologies. Overall, the Idylla™ platform showed a concordance rate with IHC of 98.0% for microsatellite stable (MSS)/proficient MMR (pMMR) cases and 81.8% for MSI/deficient MMR (dMMR) cases. The TapeStation 4200 system showed a concordance rate with IHC of 96.0% for MSS/pMMR cases and 45.4% for MSI/dMMR cases. The concordance rates of the TapeStation 4200 system with respect to the Idylla™ platform were 98.1% for MSS profile and 57.8% for MSI profile. Discordant cases were analyzed using the Titano MSI kit. Considering pre-analytical factors, no significant variation in concordance rate among IHC analyses and molecular systems was observed by considering the presence of an acellular mucus cut-off >50% of the tumor area, FFPE year preparation, and DNA concentration. Conversely, the Idylla™ platform showed a significant variation in concordance rate with the IHC approach by considering a neoplastic cell percentage >50% (*p*-value = 0.002), and the TapeStation 4200 system showed a significant variation in concordance rate with the IHC approach by considering a DNA integrity number (DIN) ≥4 as cut-off (*p*-value = 0.009). Our data pinpoint a central role of the pre-analytical phase in the diagnostic outcome of MSI testing in CRC.

## 1. Introduction

The DNA mismatch repair (MMR) complex is highly conserved and performs an essential role in maintaining genomic stability by recognizing and repairing short insertions, short deletions, and single-base mismatches that can arise during DNA replication and recombination [1]. Deficient MMR (dMMR) can occur due to germline and/or somatic mutations or epigenetic silencing, resulting in a significantly elevated spontaneous mutation rate (i.e., mutator phenotype) [2,3]. Germline mutation(s) of the MMR genes is the hallmark of Lynch syndrome and constitutional mismatch repair deficiency (CMMRD) [3,4]. Epigenetic silencing is usually represented by *MLH1* gene promoter hypermethylation, whereas secondary epigenetic silencing of *MSH6* is observed after neoadjuvant radio-chemotherapeutic treatments [5,6].

To date, immunohistochemical (IHC) analysis of four of the seven MMR proteins (i.e., MLH1, PMS2, MSH2, and MSH6) represents the most widely used approach for the evaluation of microsatellite instability (MSI) status, but has some disadvantages [3,7]. The original approach used to determine MSI involves the direct determination of replication errors in DNA microsatellite sequences. Microsatellites are short tandem sequences constituted by mono- or dinucleotide repetitions largely distributed in the genome in both coding and non-coding regions [8]. Microsatellites may be affected by errors in the duplication process generally restored to by the MMR complex.

MSI testing has been performed for many years using the Bethesda panel (*BAT25*, *BAT26*, *D2S123*, *D5S346*, and *D17S250*) [3] along with newer quasi-monomorphic loci [3] using polymerase chain reaction (PCR) and capillary electrophoresis and is considered the gold standard technique for the detection of MSI status. More recently, new MSI assays and instruments have been deployed in the clinical setting [9,10]. In particular, a fully automatized PCR, followed by high-resolution melt curve analysis (Idylla™, Biocartis, Mechelen, Belgium), has been reported to reliably assess MSI status with minimal hands-on time [11,12]. Additionally, the automated microfluidic electrophoretic-run chip-based assay (TapeStation 4200, Agilent Technologies, Santa Clara, CA, USA) represents an easy, fast, and low-cost alternative [13,14]. In colorectal carcinoma (CRC), MSI status is associated with a better overall prognosis in radically resected disease showing prominent intratumoral lymphocytic infiltration, frequent intratumor phenotypic heterogeneity, mucinous histology, and other rare histotypes, such as medullary carcinoma and signet-ring cell adenocarcinoma [15,16,17,18,19]. From a clinical point of view, CRC MMR screening/MSI testing has several important implications: (i) universal screening has been recommended to identify Lynch syndrome families [2,20]; (ii) stage II/III CRC cancers should be tested because dMMR/MSI status may affect decision making on adjuvant treatment [21]; (iii) dMMR/MSI tumors are eligible for immune checkpoint inhibitor therapies (KEYNOTE-177 (NCT02563002) [22]) and are characterized by an overexpression of programmed death-ligand 1 (PD-L1) [23,24,25,26]; (iv) its association with *BRAF* mutation drives additional prognostic considerations, especially in stage IV [27].

Although MSI assays have been performed in clinical practice for many years, methodological challenges are still an issue, particularly when the tumor sample is sub-optimal. Thus, it is not surprising that a recent report demonstrated that almost 10% of metastatic CRC patients had been enrolled for immunotherapy with a false-positive dMMR or MSI-PCR result assessed by local laboratories [28]. Most of these discordant results are due to pre-analytical errors [29]. Thus, the European Society for Medical Oncology (ESMO) recommends that both MMR-IHC and MSI-PCR should be performed in assessing eligibility to treatment with immune checkpoint inhibitors (ICIs) [3].

To date, the impact of DNA qualification on MSI testing results has not been investigated. To fill this knowledge gap, the concordance rates among IHC and Idylla and TapeStation 4200 were correlated to DNA quality. Discordant cases were further analyzed by the Titano MSI test (Diatech Pharmacogenetics, Jesi, Italy). Our data may be useful in better tailoring the most suitable methodology in relation to the nucleic acid features for any given sample.

## 2. Material and Methods

### 2.1. Study Design

From 2007 to 2019, 75 cases of mucinous CRC were retrospectively selected excluding those that underwent neoadjuvant treatments.

All information regarding human material was managed using anonymous numerical codes, and all samples were handled in compliance with the Declaration of Helsinki (http://www.wma.net/en/30publications/10policies/b3/).

The original hematoxylin-and-eosin (H&E)-stained sections were reviewed by two expert gastrointestinal pathologists (PPa and CC) to confirm a mucinous histotype according to the World Health Organization (WHO) 2019 classification [30].

For each patient, the site of the primary tumor (right colon, transverse, left colon, rectosigmoid), the pathological classification according to the Union for International Cancer Control (UICC) 2017 (pT, pN, pM), the presence of vascular hematic invasion (V), vascular lymphatic invasion (L), and perineural invasion (Pn), and surgical resection margins (R) status were reported (Supplementary Table S1). Moreover, neoplastic cellular percentage, the presence of necrosis, desmoplasia, and tumor-infiltrating lymphocytes (TILs) were evaluated by microscopic visual inspection by dedicated pathologists. Mucinous acellular component was categorized as absent (<1%) or present (≤50% or >50%) after the microscopic revision performed by two expert gastrointestinal pathologists (Supplementary Table S2).

**Figure 1:**
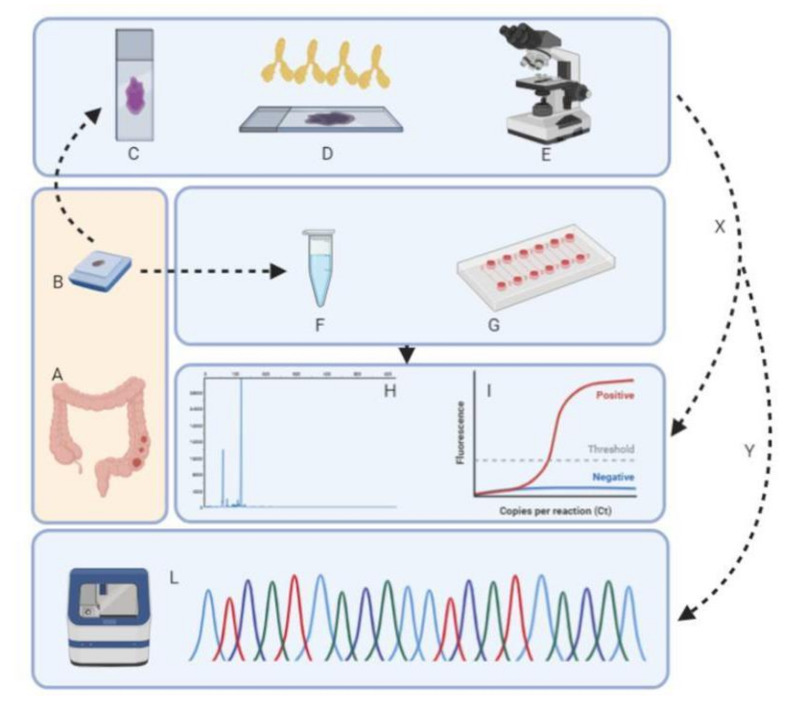
Seventy-five formalin-fixed and paraffin-embedded (FFPE) block cases of mucinous colo-rectal carcinoma (CRC) were retrospectively selected (**A**,**B**). The original hematoxylin-and-eosin (H&E)-stained sections were reviewed (**C**) to confirm the original diagnosis. Each sample underwent immunohistochemistry (IHC) in order to evaluate the mismatch repair (MMR) status (**D**,**E**). DNA was extracted and quantified from each sample from either tumor or non-neoplastic material (**F**,**G**). MSI status was assessed (**X**) by microfluidic platform (TapeStation 4200, **H**) and fully automated PCR approach (Idylla™, **I**). Discordant cases among the three techniques were further investigated (**Y**) by Sanger sequencing (Titano MSI kit, Diatech Pharmacogenetics, Jesi, Italy, **L**).

A normal tissue formalin-fixed and paraffin-embedded (FFPE) block was selected to compare tumor molecular profile with corresponding normal tissue in the microfluidic analysis. Each case was then evaluated by using the three different approaches (IHC for MMR status, microfluidic analysis, and a fully closed PCR system for MSI detection). Discordant cases were then analyzed by using the Titano MSI test on the Applied Biosystems 3130XL genetic analyzer platform (Figure 1).

### 2.2. Immunohistochemical Analysis

MMR protein analysis (MLH1, PMS2, MSH2, and MSH6) was performed by an IHC approach with the automated Autostainer Link 48 (DAKO Carpinteria, CA, USA) platform according to the manufacturer’s instructions. Briefly, 3 µm thick FFPE tissue sections were deparaffinized in xylene, rehydrated in graded alcohols, washed in double-distilled water, and pretreated with DAKO solution (EnVision FLEX Target Retrieval Solution, High pH 50×) at 97 °C. The slides were incubated with primary monoclonal antibodies against MLH1 (clone ES05 diluted 1:50, DAKO), PMS2 (clone EP51 diluted 1:40, DAKO), MSH2 (clone FE11 diluted 1:50, DAKO), MSH6 (clone EP49 diluted 1:50, DAKO) for 30 min. Antigen–antibody reaction was visualized using the EnVision FLEX kit with diaminobenzidine as chromogen; slides were counterstained with hematoxylin and, finally, covered.

The evaluation of IHC results was blindly and independently performed by two expert pathologists (PPa and CC). MMR protein expression was categorized as (i) retained (i.e., proficient MMR; pMMR), when a moderate to strong expression (similar to what is observed in the stromal cells as internal control) was present in ≥10% of tumor cells; (ii) lost (i.e., dMMR), in case of complete loss of nuclear expression in cancer cells; and (iii) indeterminate, if IHC staining intensity in tumor cells was lower than the internal control or the tumor was positive in <10% [31]. Absence of immunoreactions in the neoplastic area and internal controls were classified as “inadequate for IHC evaluation sample”.

### 2.3. DNA Extraction and Qualification

For each patient, four slides of 5 micron from the tumor tissue and from the corresponding normal mucosa were used. After manual microdissection of the neoplastic cell area, DNA was extracted by using the Mini Amp kit (Qiagen, Hilden, Germany) following the manufacturer’s instructions. Finally, DNA was eluted in 30 µL of DNAse and RNAse-free water (Thermo Fisher Scientifics, Waltham, MA, USA) and qualified on the TapeStation 4200 microfluidic platform by using a genomic ladder and buffer (Agilent Genomic ScreenTape, Agilent Technologies) on the Genomic ScreenTape device (Agilent Technologies), following the manufacturer’s instructions. The extracted and qualified DNA was used for MSI analysis and stored for confirmation in case of discordant results. Equivalent serial FFPE sections were used for Idylla™ analysis as reported in the specific section.

### 2.4. Microfluidic Analysis for MSI Status Evaluation

The PCR was performed to amplify genomic DNA by adopting primers from the Bethesda panel. An amount of 20 ng was considered adequate for PCR amplification. MSI evaluation was realized by running 1 µL of amplified product from both tumor and normal tissues of each reaction mix for all the patients. Briefly, 3 µL of D1000 Buffer (Agilent Technologies) and 1 µL for each PCR product were automatically charged on a solid device constituted by 16 nanocapillaries (D1000 ScreenTape), where electrophoretic run was performed on the TapeStation 4200 platform. Results were inspected by using the TapeStation Analysis Software (Agilent Technologies).

### 2.5. Idylla™ MSI Assay

The fully automated Idylla™ MSI Test performed the detection of microsatellite instability directly from FFPE human cancer tissue sections utilizing a PCR reaction followed by high-resolution melting curve analysis, as specified by the manufacturer.

For each of the 75 patients, four slides of 5 micron derived from the tumor tissue were used to perform the Idylla™ MSI Test. From each slide, neoplastic cells were directly scraped on two circle papers (GE Healthcare Life Sciences Whatman) previously wet with nuclease-free water (Ambion, Thermo Fisher Scientifics) to make a sandwich. This sample-paper sandwich was directly put in the Idylla™ MSI Test cartridge. The MSI cartridge was then loaded into the Idylla™ instrument for molecular analysis. Briefly, into the cartridge, DNA was extracted after tissue homogenization and cell lysis performed by a combination of HIFU, enzymatic/chemical digestion, and heat. The extracted DNA was then transported into five PCR chambers for amplification. The Idylla™ MSI Test analyzes homopolymers in seven biomarkers (*ACVR2A*, *BTBD7*, *DIDO1*, *MRE11*, *RYR3*, *SEC31A,* and *SULF2*) by adopting fluorescently labeled molecular beacon probes. Results were carried out by an automatic specific software able to detect a minimum allele frequency of 10%, calculating a probability score (MSI score) for all the tested biomarkers. The obtained results were considered valid if ≥5 out of the 7 MSI biomarkers showed valid profiles. In particular, after this quality check, the tested samples were classified as MSI-high (MSI-H) when 2≥ of the 7 biomarkers were mutated, and microsatellite stable (MSS) when <2 of the 7 biomarkers were mutated.

### 2.6. Titano MSI Test

Discordant samples between IHC and molecular approaches were further analyzed by adopting the Titano MSI test (Diatech Pharmacogenetics). Briefly, the extracted DNA of the tumor and the corresponding normal mucosa were analyzed with the MSI Titano kit following the manufacturer’s instructions. The Titano MSI kit allows the determination of microsatellite instability status in colorectal cancer samples by multiplex amplification with fluorescent primers and subsequent DNA fragment analysis on an automated sequencer. Starting from 20 ng of the extracted DNA, this tool is able to detect variation in the number of microsatellite loci of 10 different molecular targets (*BAT25*, *BAT26*, *D2S123*, *D17S250*, *D5S346*, *BAT40*, *D18S58*, *NR21*, *NR24*, and *TGFβRII*) by comparing peak profiles generated from the capillary electrophoresis run of the tumor and the corresponding normal tissue samples for each patient.

## 3. Results

Seventy-three (73/75; 97.3%) of the selected CRCs were successfully analyzed by the three technical approaches. Two cases showed an inadequate result for IHC analysis.

A median DNA concentration of 20.6 ng/µL (ranging from 0.70 to 73.5 ng/µL) for the tumor samples and a median DNA concentration of 11.8 ng/µL (ranging from 0.12 to 60.0 ng/µL) for the normal samples were obtained.

IHC analysis showed a pMMR and a dMMR status in 51 (51/73; 69.9%) and 22 (22/73; 30.1%) cases, respectively. The Idylla™ platform and TapeStation 4200 system were able to identify an MSS profile in 54 (54/73; 74.0%) and 61 (61/73; 83.5%) cases, respectively, whereas an MSI profile was reported in 19 (26.1%) and 12 (16.4%) cases, respectively.

In particular, the Idylla™ platform showed a concordance rate with IHC of 98.0% (50/51) for the MSS/pMMR cases and 81.8% (18/22) for the MSI/dMMR cases (k = kappa = 0.83; 95% C.I.: 0.69 to 0.97). The TapeStation 4200 system showed a concordance rate with IHC of 96.0% (49/51) for the MSS/pMMR cases and 45.4% (10/22) for the MSI/dMMR cases (k = 0.48; 95% C.I.: 0.25 to 0.7). The concordance rate of the TapeStation 4200 system with respect to the Idylla™ platform was 98.1% (53/54) for the MSS profile and 57.8% (11/19) for the MSI profile. The results are summarized in Table 1 and Supplementary Table S3.

### 3.1. MMR and MSI Status on Acellular Mucin

No significant variation in concordance rate among IHC analyses and molecular systems was observed by considering the presence of an acellular mucus cut-off >50% of the tumor area. The Idylla™ platform and TapeStation 4200 system showed a concordance rate with respect to the IHC approach of 100.0% (23/23) and 95.6% (22/23) for MSS evaluation and 80.0% (8/10) and 30.0% (3/10) for MSI detection, respectively (k = 0.85; 95% C.I.: 0.65 to 1; k = 0.31; 95% C.I.: −0.03 to 0.64), when considering a mucinous component cut-off ≤50%; whereas a concordance rate with respect to the IHC approach of 96.4% (27/28) for MSS evaluation and 91.6% (10/12) and 58.3% (7/12) for MSI detection, respectively (k = 0.82; 95% C.I.: 0.62 to 1; k = 0.61; 95% C.I.: 0.33 to 0.88), were evaluated by considering a mucinous component cut-off >50% (*p*-value = 0.836, 0.182). The results are summarized in Supplementary Table S4.

### 3.2. Sample Stratification by Year

Statistical evaluation also did not reveal any significant variation in concordance rate among IHC analyses and molecular systems by considering the FFPE year preparation. The Idylla™ platform and TapeStation 4200 system both showed a concordance rate with respect to the IHC approach of 100.0% (38/38) for MSS evaluation and 73.4% (11/15) and 26.7% (4/15) for MSI detection, respectively (k = 0.8; 95% C.I.: 0.61 to 0.98; k = 0.34; 95% C.I.: 0.08 to 0.6), by considering FFPE samples from 2007–2017, while a concordance rate with respect to the IHC approach of 92.3% (12/13) and 84.6% (11/13) for MSS evaluation and 100.0% (7/7) and 85.7% (6/7) for MSI detection, respectively (k = 0.89; 95% C.I.: 0.69 to 1; k = 0.68; 95% C.I.: 0.35 to 1), were evaluated by considering FFPE samples from 2017–2019 (*p*-value = 0.537, 0.136). The results are summarized in Supplementary Table S5.

### 3.3. MMR and MSI Status on Neoplastic Cells

The Idylla™ platform showed a significant variation in concordance rate with the IHC approach by considering the neoplastic cell percentage (*p*-value = 0.002). In this setting, Idylla™ showed a concordance rate with respect to IHC of 100.0% (17/17) for MSS evaluation and 40.0% (2/5) for MSI detection (k = 0.51; 95% C.I.: 0.06 to 0.96), by considering a neoplastic cell percentage cut-off <50%; while a concordance rate with respect to the IHC approach of 97.0% (33/34) for MSS evaluation and 94.1% (16/17) for MSI detection (k = 0.91; 95% C.I.: 0.79 to 1) were evaluated by considering a neoplastic cell percentage cut-off >50%. The TapeStation 4200 system did not highlight any statistically relevant differences by evaluating the IHC concordance rate by inspecting the cell percentage cut-off (*p*-value = 0.142). The results are summarized in Table 2.

### 3.4. MMR and MSI Status Based on DNA Quantity and Quality

No significant variation in concordance rate among IHC analyses and molecular systems was observed by considering DNA concentration. The Idylla™ platform and TapeStation 4200 system both showed a concordance rate with respect to the IHC approach of 100.0% (36/36) and 97.2% (35/36) for MSS evaluation and 78.9% (15/19) and 52.6% (10/19) for MSI detection, respectively (k = 0.83; 95% C.I.: 0.67 to 0.99; k = 0.55; 95% C.I.: 0.32 to 0.79), by considering a DNA concentration cut-off ≤25 ng; while a concordance rate with respect to the IHC approach of 93.3% (14/15) for MSS evaluation and 91.6% (10/12) and 100.0% (3/3) and 0.0% (0/3) for MSI detection, respectively (k = 0.82; 95% C.I.: 0.49 to 1; k = −0.09; 95% C.I.: −0.23 to 0.05), were evaluated by considering a DNA concentration cut-off >25 ng (*p*-value = 0.969, 0.054). The results are summarized in Supplementary Table S6.

The TapeStation 4200 system showed significant variation in concordance rate with the IHC approach by considering a DNA integrity number (DIN) ≥4 as cut-off (*p*-value = 0.009). In particular, this approach revealed a concordance rate with IHC of 96.5% (28/29) for MSS evaluation and 23.1% (3/13) for MSI detection (k = 0.24; 95% C.I.: −0.04 to 0.52) by considering a DIN <4; while a concordance rate with respect to the IHC approach of 95.2% (20/21) for MSS evaluation and 87.5% (7/8) for MSI detection (k = k = 0.83; 95% C.I.: 0.6 to 1) were evaluated by considering a DIN ≥4. The Idylla™ system did not highlight any statistically relevant differences by evaluating the IHC concordance rate by inspecting the DIN cut-off (*p*-value = 0.285). The results are summarized in Table 3.

### 3.5. Analysis of Discordant Samples due to Low Quality of DNA Integrity

Taking into account a DIN < 4 as cut-off, 11 out of 73 (15.1%) cases were globally discordant by comparing the TapeStation 4200 system with respect to the IHC approach and the Idylla™ platform. Each sample was successfully analyzed by applying the Titano MSI kit. The results showed that in 1 out of 11 (9.1%) cases, the TapeStation 4200 system and Titano kit detected MSI and MSS profiles, respectively; in 63.6% (7/11) of cases, the TapeStation 4200 system and Titano kit detected MSS and MSI profiles, respectively, while in 27.3% (3/11), both techniques identified MSS profile. As far as the comparison between Idylla™ and the Titano MSI kit is concerned, overall, 10 concordant cases (*n* = 3 MSS and *n* = 7 MSI) were detected. Only one discordant case (Idylla™ MSI-H/Titano MSI kit MSS) was reported. Taking into account the IHC results, eight concordant cases (*n* = 1 MSS and *n* = 7 dMMR/MSI) were reported. Overall, three discordant cases (MSS for Titano MSI kit analysis and dMMR for IHC evaluation) were detected. The results are summarized in Table 4.

## 4. Discussion

MMR/MSI testing has acquired a key role in CRC patient management [3,23,24]. Several studies have demonstrated that dMMR CRCs feature an increased expression of PD-L1 within the neoplastic component and within tumor-infiltrating lymphocytes, allowing the tumor to evade antitumor immunity. Notably, it has been proved that patients with dMMR/MSI tumors may benefit from anti-PD-1 therapy; similar results were reported in a refractory MSI metastatic CRC (mCRC) patient setting, with 31.3% of the patients achieving an investigator-assessed objective response and 69% of the patients having disease for 12 weeks or longer [23]. Consistent with such preliminary data, a recent phase III study showed a significantly higher progression-free survival (PFS) with pembrolizumab versus standard chemotherapy plus or minus biological agents in patients with MSI-H mCRC [22]. Thus, dMMR/MSI-H profile is nowadays validated as a predictive biomarker for immune checkpoint inhibitors (ICIs) [22].

Given the importance of MSI testing in clinical practice, several assays have been investigated and validated, but careful attention should be paid to the interpretation of results in light of their specificity and sensitivity, as well as potential intrinsic and analytical limitations. First of all, IHC is limited by the subjectivity of interpretations. Interestingly, it has been shown that pMMR IHC profile may be retained in the presence of MSI-H status in cases associated with germline MMR gene mutations leading to antigenically intact, non-functioning proteins [32]. These cases typically lack association with sporadic *MLH1* hypermethylation. On the contrary, it has been shown that multiplex PCR may be associated with false-positive results in the presence of specific variants mimicking unstable alleles that are outside the quasi-monomorphic range [28]. Currently, most trials investigating ICIs in MSI-H cancers adopt as an inclusion criterion the presence of locally assessed MSI or dMMR status using only one diagnostic method (PCR or IHC). However, it has been suggested that some patients with MSI-H mCRC and early progressive disease at first radiological re-assessment may have been affected by MSS/pMMR cancers rather than primarily ICI-resistant MSI-H ones [28]. Therefore, optimal adoption of MSI testing assays with at least two concordant results may be crucial in selecting patients with MSI-H cancers for immunotherapy, in order to maximize the efficacy of this therapeutic option. Indeed, in the KEYNOTE-177 first-line registration trial [22], the subgroup of patients showing greater PFS benefit from standard chemotherapy compared with pembrolizumab included patients with MSS/pMMR cancers, and therefore, central re-assessment of MSI status is eagerly awaited as post hoc analysis of the trial. The results of such analyses may shed further light on the importance of the standardization of MSI testing prior to immunotherapy decisions in gastrointestinal oncology.

A high concordance rate between MMR-IHC testing and PCR-based MSI testing has been described [33]. However, both methods are affected by intrinsic pre-analytical and interpretative biases, and as a result, almost 10% of metastatic CRC patients that were enrolled for immunotherapy had a false-positive dMMR or MSI-PCR result assessed by local laboratories [28]. In this frame, ESMO has recently recommended that both MMR-IHC and MSI-PCR should be performed to assess eligibility to treatment with ICIs [3]. No study considered the impact of DNA qualification on MSI testing, particularly in mucinous CRC. This entity is a relatively frequent CRC subtype [19], which is affected by low tumor cellularity that may have an impact on the downstream molecular characterization.

We analyzed a relatively large series of mucinous CRCs by two different MSI methods. No statistically relevant discrepancies were observed in the concordance rate between the two molecular platforms and the IHC approach. In particular, a higher concordance rate was globally observed between IHC and the Idylla™ system (93.1%) with respect to the TapeStation 4200 platform (80.2%). The main discrepancies between IHC testing and MSI testing were identified in false-negative results (4/22, 18.2%, and 12/22, 54.5%, respectively). Focusing on specific morphological features of mucinous CRC samples, no statistically relevant analysis was highlighted by inspecting year for the sample preparation, DNA concentration, or mucous component. On the other hand, we demonstrated that parameters such as neoplastic cellularity and DIN may influence the analytical performance of the Idylla™ system and TapeStation 4200 platform. In particular, a minimum input of 50% of neoplastic cells, selected as a cut-off for comparative analysis following the Italian guidelines for CRC molecular analysis (https://www.aiom.it/wp-content/uploads/2019/10/2019_LG_AIOM_Colon-1.pdf), is fundamental to adequately perform molecular analysis by using the Idylla™ system. This condition may not be reached in small biopsy samples or post-radiotherapy patients where contamination with non-neoplastic elements may reduce tumor cell percentage. A DIN < 4 significantly affected TapeStation 4200 results. This method is based on a crucial amplification step where primer length (~200 bp) may not be able to amplify highly fragmented DNA generally derived from over-fixation in the analytical management phase of routine diagnostic samples. To better explain the last limitation, 11 discordant samples between IHC and the TapeStation 4200 platform were re-analyzed by an orthogonal approach. We observed that the Titano MSI kit showed a technical performance similar to the TapeStation 4200 system, and this is mostly due to the comparable amplicon lengths derived from these two methods. Moreover, four MSI samples revealed by the Titano MSI kit were considered as MSS by using the TapeStation 4200 system, but in relation to this point, all these cases showed an MSI-low status (MSI-L) by adopting the TapeStation 4200 approach, with a single unstable locus. Notably, the term MSI-L should be abandoned, and MSI-L tumors should be considered as MSS tumors [9]. These data suggest that the main difference between the TapeStation 4200 and Titano systems is the number of microsatellites analyzed, since TapeStation 4200 is considering 5 probes and Titano 10. Thus, the increasing number of molecular target and a high quality of starting DNA may significantly contribute to considering MSI molecular analysis as a valid diagnostic approach for MSI testing in routine practice. The current MSI testing is based on two main panels: (i) five microsatellites comprising two mononucleotide repeats (*BAT25* and *BAT26*) and three dinucleotide repeats (*D5S346*, *D2S123*, and *D17S250*) (i.e., the Bethesda panel), and (ii) five poly-A mononucleotide repeats (*BAT25*, *BAT26*, *NR21*, *NR24*, and *NR27*). The pentaplex panel of the five poly-A mononucleotide repeats has been recommended given its higher sensitivity and specificity [10]. Moreover, it may obviate the need for normal tissue for comparison, which is of central importance in the analysis of small biopsies obtained from cancer tissue.

Overall, these data pinpointed a central role of the pre-analytical phase in the diagnostic outcome of MSI testing in CRC. Future efforts should focus on the definition of the required sample and DNA qualification to ensure an adequate evaluation of MSI status in CRC.

## Figures and Tables

**Table 1:** Overall data obtained by comparing the results between IHC and TapeStation 4200, IHC and Idylla™, and Idylla™ and TapeStation 4200.

**IHC vs. Tape Station 4200**			
TapeStation 4200	IHC		
	dMMR	pMMR	Total
MSI-H	10 (13.7)	2 (2.7)	12 (16.4)
MSS	12 (16.4)	49 (67.1)	61 (83.6)
Total	22 (30.1)	51 (69.9)	73 (100.0)
k = 0.48; 95% C.I.: 0.25 to 0.7.			
**IHC vs. Idylla™**			
Idylla™	IHC		
	dMMR	pMMR	Total
MSI-H	18 (24.6)	1 (1.4)	19 (26.0)
MSS	4 (5.5)	50 (68.5)	54 (74.0)
Total	22 (30.1)	51 (69.9)	73 (100.0)
k = 0.83; 95% C.I.: 0.69 to 0.97.			
**Idylla™ vs. Tape Station 4200**			
TapeStation 4200	Idylla™		
	MSI-H	MSS	Total
MSI-H	11 (15.0)	1 (1.4)	12 (16.4)
MSS	8 (11.0)	53 (72.6)	61 (83.6)
Total	19 (26.0)	54 (74.0)	73 (100.0)
k = 0.64; 95% C.I.: 0.43 to 0.85.			

Abbreviations: dMMR: deficient mismatch repair; IHC: immunohistochemistry; MSI-H: high microsatellite instability; pMMR: proficient mismatch repair.

**Table 2:** Data obtained by comparing the results between IHC and TapeStation 4200, IHC and Idylla™, and Idylla™ and TapeStation 4200, taking into account the percentage of neoplastic cells.

**≤50% Neoplastic Cells IHC vs. TapeStation 4200**			
TapeStation 4200	IHC		Total
	dMMR	pMMR	
MSI-H	1 (4.5)	0 (0.0)	1 (4.5)
MSS	4 (18.2)	17 (77.3)	21 (95.5)
Total	5 (22.7)	17 (77.3)	22 (100.0)
k = 0.28; 95% C.I.: −0.16 to 0.72.			
**>50% Neoplastic Cells IHC vs. Tape Station 4200**			
TapeStation 4200	IHC		Total
	dMMR	pMMR	
MSI-H	9 (17.6)	2 (3.9)	11 (21.6)
MSS	8 (15.7)	32 (62.7)	40 (78.4)
Total	17 (33.3)	34 (66.7)	51 (100.0)
k = 0.52; 95% C.I.: 0.26 to 0.77; *p*-value for difference between k: 0.142.			
**≤50% Neoplastic Cells IHC vs. Idylla™**			
Idylla™	IHC		Total
	dMMR	pMMR	
MSI-H	2 (9.1)	0 (0.0)	2 (9.1)
MSS	3 (13.6)	17 (77.3)	20 (90.9)
Total	5 (22.7)	17 (77.3)	22 (100.0)
k = 0.51; 95% C.I.: 0.06 to 0.96.			
**>50% Neoplastic Cells IHC vs. Idylla™**			
Idylla™	IHC		Total
	dMMR	pMMR	
MSI-H	16 (31.4)	1 (2.0)	17 (33.3)
MSS	1 (2.0)	33 (64.7)	34 (66.7)
Total	17 (33.3)	34 (66.7)	51 (100.0)
k = 0.91; 95% C.I.: 0.79 to 1; *p*-value for difference between k: 0.002.			
**≤50% Neoplastic Cells Idylla™ vs. TapeStation 4200**			
TapeStation 4200	Idylla™		Total
	MSI-H	MSS	
MSI-H	1 (4.2)	0 (0.0)	1 (4.2)
MSS	1 (4.2)	22 (91.7)	23 (95.8)
Total	2 (8.3)	22 (91.7)	24 (100.0)
k = 0.65; 95% C.I.: 0.01 to 1.			
**>50% Neoplastic Cells Idylla™ vs. TapeStation 4200**			
TapeStation 4200	Idylla™		Total
	MSI-H	MSS	
MSI-H	10 (19.6)	1 (2.0)	11 (21.6)
MSS	7 (13.7)	33 (64.7)	40 (78.4)
Total	17 (33.3)	34 (66.7)	51 (100.0)
k = 0.61; 95% C.I.: 0.38 to 0.85; *p*-value for difference between k: 0.913.			

Abbreviations: dMMR: deficient mismatch repair; IHC: immunohistochemistry; MSI-H: high microsatellite instability; pMMR: proficient mismatch repair.

**Table 3:** Data obtained by comparing the results between IHC and TapeStation 4200, IHC and Idylla™, and Idylla™ and TapeStation 4200, taking into account the DIN.

**DIN < 4 IHC vs. TapeStation 4200**			
TapeStation 4200	IHC		Total
	dMMR	pMMR	
MSI-H	3 (7.1)	1 (2.4)	4 (9.5)
MSS	10 (23.8)	28 (66.7)	38 (90.5)
Total	13 (31.0)	29 (69.0)	42 (100.0)
k = 0.24; 95% C.I.: −0.04 to 0.52.			
**DIN ≥ 4 IHC vs. TapeStation 4200**			
TapeStation 4200	IHC		Total
	dMMR	pMMR	
MSI-H	7 (24.1)	1 (3.4)	8 (27.6)
MSS	1 (3.4)	20 (69.0)	21 (72.4)
Total	8 (27.6)	21 (72.4)	29 (100.0)
k = 0.83; 95% C.I.: 0.6 to 1; *p*-value for difference between k: 0.009.			
**DIN < 4 IHC vs. Idylla™**			
Idylla™	IHC		Total
	dMMR	pMMR	
MSI-H	9 (21.4)	0 (0.0)	9 (21.4)
MSS	4 (9.5)	29 (69.0)	33 (78.6)
Total	13 (31.0)	29 (69.0)	42 (100.0)
k = 0.76; 95% C.I.: 0.54 to 0.98.			
**DIN ≥ 4 IHC vs. Idylla™**			
Idylla™	IHC		Total
	dMMR	pMMR	
MSI-H	8 (27.6)	1 (3.4)	9 (31.0)
MSS	0 (0.0)	20 (69.0)	20 (69.0)
Total	8 (27.6)	21 (72.4)	29 (100.0)
k = 0.92; 95% C.I.: 0.76 to 1; *p*-value for difference between k: 0.285			
**DIN < 4 Idylla™ vs. TapeStation 4200**			
TapeStation 4200	Idylla™		Total
	MSI-H	MSS	
MSI-H	3 (7.1)	1 (2.4)	4 (9.5)
MSS	6 (14.3)	32 (76.2)	38 (90.5)
Total	9 (21.4)	33 (78.6)	42 (100.0)
k = 0.38; 95% C.I.: 0.03 to 0.73.			
**DIN ≥ 4 Idylla™ vs. TapeStation 4200**			
TapeStation 4200	Idylla™		Total
	MSI-H	MSS	
MSI-H	8 (27.6)	0 (0.0)	8 (27.6)
MSS	1 (3.4)	20 (69.0)	21 (72.4)
Total	9 (31.0)	20 (69.0)	29 (100.0)
k = 0.92; 95% C.I.: 0.76 to 1; *p*-value for difference between k: 0.012			

Abbreviations: DIN: DNA integrity number; dMMR: deficient mismatch repair; IHC: immunohistochemistry; MSI-H: high microsatellite instability; pMMR: proficient mismatch repair.

**Table 4:** Comparative analysis in the discordant cases.

Titano	IHC	Idylla™	TapeStation 4200
MSS	pMMR	MSS	MSI-H
MSI	dMMR	MSI-H	MSS *
MSI	dMMR	MSI-H	MSS
MSI	dMMR	MSI-H	MSS
MSS	dMMR	MSS	MSS
MSS	dMMR	MSS	MSS
MSS	dMMR	MSI-H	MSS
MSI	dMMR	MSI-H	MSS *
MSI	dMMR	MSI-H	MSS *
MSI	dMMR	MSI-H	MSS *
MSI	dMMR	MSI-H	MSS *

Note: * MSI-L. Abbreviations: dMMR: deficient mismatch repair; IHC: immunohistochemistry; MSI-H: high microsatellite instability; MSI-L: low microsatellite instability; MSS: microsatellite stable; pMMR: proficient mismatch repair.

## Supplementary Materials

The following are available online at https://www.mdpi.com/2073-4409/9/9/2019/s1, Table S1. Patients characteristics. For each patient, the site of the primary tumor (right colon, transvers, left colon, rectosigmoid), the pathological classification according to the Union for International Cancer Control (UICC) 2017 (pT, pN, pM), the presence of vascular hematic invasion (V), vascular lymphatic invasion (L), and perineural invasion (Pn), were reported. Table S2. Patients characteristics. For each patient, neoplastic cellular percentage, the presence of necrosis, desmoplasia, inflammation and mucinous acellular component, were reported. Table S3. Summary of molecular analysis of MMR/MSS status for 73 mucinous-CRC patients. Table S4. Data obtained by comparing results between IHC and TapeStation 4200, IHC and Idylla™, and Idylla™ and TapeStation 4200, taking into account the percentage of acellular mucin. Table S5. Data obtained by comparing results between IHC and TapeStation 4200, IHC and Idylla™, and Idylla™ and TapeStation 4200, taking into account the stratification by years. Table S6. Data obtained by comparing results between IHC and TapeStation 4200, IHC and Idylla™, and Idylla™ and TapeStation 4200, taking into account DNA concentration.
