# Supplementary material for: Impact of Pre-Analytical Factors on MSI Test Accuracy in Mucinous Colorectal Adenocarcinoma: A Multi-Assay Concordance Study
Source: Cells. 2020 Sep 2;9(9):2019. doi: 10.3390/cells9092019 (PMC7565496; doi:10.3390/cells9092019)
Supplement: Supplementary file 1 [file cells-09-02019-s001.pdf]

cells MDPI

Supplementary Table S1. Patients' characteristics. For each patient, the site of the primary tumor (right colon, transvers, left colon, rectosigmoid), the pathological classification according to the Union for International Cancer Control (UICC) 2017 (pT, pN, pM), the presence of vascular hematic invasion (V), vascular lymphatic invasion (L), and perineural invasion (Pn), were reported.

Patient	Site	pTNM	V, L, Pn
1	transvers	pT2 pN0	V0 L0 Pn0
2	right colon	pT3 pN0	V1 L1 Pn0
3	left colon	pT4a pN2a	V0 L1 Pn0
4	right colon	pT4a pN0	V1 L1 Pn0
5	left colon	pT3 pN0	V0 L1 Pn0
6	left colon	pT4a pN0	V0 L0 Pn0
7	right colon	pT3 pN0	V0 L0 Pn0
8	right colon	pT3 pN0	V0 L0 Pn0
9	right colon	pT4 pN0	V0 L0 Pn0
10	right colon	pT3 pN0	V0 L0 Pn0
11	right colon	pT4a pN1b	V0 L1 Pn0
12	right colon	pT4a pN2b	V1 L1 Pn1
13	left colon	pT3 pN0	V0 L0 Pn0
14	left colon	pT3 pN2a	V0 L1 Pn0
15	left colon	pT3 pN1b	V0 L1 Pn0
16	right colon	pT4b pN1c pM1	V0 L1 Pn0
17	left colon	pT4a pN1	V0 L1 Pn0
18	transvers	pT3 pN1b	V0 L1 Pn0
19	left colon	pT3 pN0	V0 L1 Pn0
20	right colon	pT4a pN1a pM1c	V0 L1 Pn0
21	right colon	pT3 pN0	V0 L0 Pn0
22	right colon	pT4 pN0	V0 L0 Pn0
23	rectosigmoid	pT2 pN1a	V1 L1 Pn1
24	rectosigmoid	pT4a pN1b	V1 L1 Pn0
25	right colon	pT4a pN0	V0 L0 Pn0
26	right colon	pT2 pN1a	V0 L1Pn0
27	right colon	pT3 pN0	V0 L0 Pn0
28	right colon	pT4a pN2a	V0 L1Pn0
29	transvers	pT4a(m-2)pN1aM1c	V0 L1 Pn1
30	rectosigmoid	pT3 pN2a	V1 L1 Pn1
31	right colon	pT4a pN1c	V0 L0 Pn0
32	rectosigmoid	pT3 pN0	V0 L0 Pn0
33	right colon	pT4a pN2a pM1a	V1 L1 Pn1
34	rectosigmoid	pT4a pN2b	V1 L1 Pn1
35	right colon	pT4a pN0	V0 L0 Pn0
36	left colon	pT3 pN1a	V0 L1 Pn0
37	right colon	pT2 pN0	V0 L0 Pn0
38	right colon	pT4a pN2a	V1 L1 Pn0
39	left colon	pT3a pN2a	V1 L1 Pn1
40	left colon	pT3 pN2b	V1 L1 Pn0
41	right colon	pT4a pN2a pM1b	V0 L1 Pn0
42	right colon	pT3 pN0	V0 L0 Pn0
43	right colon	pT4a pN2b	V1 L1 Pn0
44	right colon	pT4a pN2b	V1 L1 Pn0
45	right colon	pT3 pN0	V0 L0 PN0
46	right colon	pT4a pN0	V0 L0 PN0
47	right colon	pT3 pN2b	V0 L1 Pn0
48	right colon	pT1 N0	V0 L0 Pn0
49	right colon	pT3 N0	V0 L0 Pn0

50	left colon	pT3 pN1b	V0 L1 Pn0
51	rectosigmoid	pT3 pN1	V0 L1 Pn0
52	right colon	pT3 pN1	V0 L1 Pn0
53	right colon	pT4a pN1a	V1 L1 Pn1
54	right colon	pT3 pN2b	V1 L1 Pn0
55	rectosigmoid	pT3 pN0	V0 L0 Pn0
56	rectosigmoid	pT4b pN0	V0 L1 Pn0
57	left colon	pT3 pN2a pM1a (m-l)	V1L1Pn0
58	right colon	pT3 pN2a pM1a	V1L1Pn0
59	right colon	pT3 pN1a	V1 L0 Pn0
60	right colon	pT3 pN1b	V0 L1 Pn0
61	right colon	pT3 pN1b pM1a l	V1 L1 pN0
62	left colon	pT3 pN2a	V0 L1 Pn0
63	right colon	pT3 pN1	V0 L1 Pn0
64	right colon	pT3 pN0	V0 L0 Pn0
65	right colon	pT3 pN0	V0 L0 Pn0
66	right colon	pT2 pN0	V0 L0 Pn0
67	rectosigmoid	pT3 pN0	V0 L0 Pn0
68	right colon	pT3 pN0	V0 L0 Pn0
69	right colon	pT3 pN1a	V1 L0 Pn0
70	right colon	pT3 pN0	V0 L0 Pn0
71	right colon	pT3 pN1a	V1 L1 Pn0
72	right colon	pT2 (m-2)pN0	V0 L0 Pn0
73	right colon	pT3 pN1a	V0 L1 Pn0
74	right colon	pT4b pN1c pM1c	V1 L1 Pn0
75	right colon	pT3 pN1a	V0 L1 Pn0

Supplementary Table S2. Patients characteristics. For each patient, neoplastic cellular percentage, the presence of necrosis, desmoplasia, inflammation and mucinous acellular component, were reported.

Patient	% neoplastic cells	Necrosis	Desmoplasia	Inflammation	Mucin
1	95%	0%	0%	0%	>50%
2	70%	0%	<50%	<50%	<50%
3	70%	<50%	<50%	0%	>50%
4	60%	<50%	<50%	0%	>50%
5	50%	<50%	>50%	<50%	<50%
6	15%	<50%	<50%	>50%	>50%
7	60%	0%	<50%	<50%	<50%
8	60%	0%	<50%	<50%	>50%
9	60%	<50%	<50%	<50%	>50%
10	70%	<50%	<50%	<50%	<50%
11	50%	0%	0%	<50%	>50%
12	70%	0%	0%	0%	>50%
13	70%	<50%	<50%	<50%	<50%
14	50%	0%	<50%	<50%	>50%
15	70%	<50%	<50%	<50%	>50%
16	80%	<50%	<50%	<50%	>50%
17	50%	>50%	<50%	<50%	>50%
18	25%	<50%	>50%	<50%	>50%
19	75%	0%	0%	0%	<50%
20	50%	<50%	<50%	<50%	>50%
21	90%	0%	<50%	<50%	<50%
22	40%	0%	<50%	<50%	>50%
23	60%	0%	0%	0%	<50%
24	70%	0%	<50%	0%	<50%
25	30%	0%	>50%	<50%	>50%
26	90%	0%	<50%	0%	>50%
27	40%	0%	>50%	<50%	<50%
28	85%	0%	<50%	<50%	>50%
29	50%	<50%	>50%	<50%	0%
30	90%	<10%	<10%	0%	>50%
31	90%	0%	<50%	0%	>50%
32	10%	0%	>50%	<50%	>50%
33	60%	<50%	<50%	<50%	>50%
34	60%	0%	<50%	0%	>50%
35	30%	0%	<50%	<50%	>50%
36	30%	0%	<50%	<50%	50%
37	85%	<50%	<50%	<50%	<50%
38	70%	0%	>50%	<50%	<50%
39	80%	<50%	<50%	<50%	<50%
40	65%	0%	<50%	<50%	<50%
41	35%	0%	<50%	<50%	>50%
42	60%	0%	<50%	<50%	<50%
43	40%	0%	<50%	0	>50%
44	80%	>50%	<50%	0	<50%
45	80%	<50%	<50%	0	<50%
46	70%	0%	<50%	0	>50%
47	60%	0%	<50%	0	>50%
48	70%	0%	<50%	0	<50%
49	80%	0%	<50%	<50%	>50%
50	40%	0%	<50%	<50%	<50%
51	80%	0%	<50%	0	<50%
52	90%	<50%	<50%	<50%	<50%
53	90%	<50%	<50%	0	<50%
54	80%	0%	<50%	0	<50%

55	70%	0%	<50%	0	<50%
56	20%	0%	<50%	0	>50%
57	50%	0%	<50%	<50%	>50%
58	75%	0%	<50%	0%	>50%
59	40%	<50%	<50%	<50%	<50%
60	40%	0%	<50%	0%	>50%
61	70%	<50%	<50%	<50%	<50%
62	75%	0%	<50%	<50%	>50%
63	80%	<50%	0%	<50%	<50%
64	65%	0%	0%	<50%	<50%
65	60%	<50%	<50%	<50%	<50%
66	40%	<50%	<50%	<50%	>50%
67	65%	0%	<50%	<50%	>50%
68	70%	<50%	<50%	<50%	>50%
69	75%	0%	<50%	<50%	<50%
70	70%	0%	0%	<50%	>50%
71	85%	0%	0%	0%	<50%
72	75%	<50%	0%	<50%	>50%
73	50%	<50%	0%	<50%	>50%
74	80%	<50%	0%	<50%	<50%
75	50%	>50%	<50%	>50%	>50%

Supplementary Table S3. Summary of molecular analysis of MMR/MSS status for 73 mucinous-CRC patients.

ID	% Neoplastic cells	DNA amount (ng/µL)	DIN	IHC	Idylla™	TapeStation 4200
1	95%	9.7	4.0	dMMR	MSI-H	MSI-H
2	70%	10.2	2.6	dMMR	MSI-H	MSI-H
3	70%	14.2	2.9	pMMR	MSS	MSS
4	60%	8.6	2.6	dMMR	MSI-H	MSI-H
5	50%	12.3	4.7	pMMR	MSS	MSS
6	15%	0.7	NA	pMMR	MSS	MSS
7	60%	3.2	3.5	pMMR	MSS	MSS
8	60%	3.2	4.4	dMMR	MSI-H	MSI-H
9	60%	5.2	4.1	dMMR	MSI-H	MSI-H
10	70%	5.3	3.0	dMMR	MSI-H	MSS*
11	50%	10.0	3.5	pMMR	MSS	MSS
12	70%	5.1	5.4	dMMR	MSI-H	MSI-H
13	70%	4.7	2.9	dMMR	MSI-H	MSI-H
14	50%	3.7	4.6	dMMR	MSI-H	MSI-H
15	70%	3.8	4.1	dMMR	MSI-H	MSI-H
16	80%	32.9	5.6	pMMR	MSI-H	MSI-H
17	50%	25.7	3.4	pMMR	MSS	MSS
18	25%	3.0	5.7	pMMR	MSS	MSS
19	75%	20.2	4.9	pMMR	MSS	MSS*
20	50%	10.5	4.4	pMMR	MSS	MSS*
21	90%	13.2	6.1	dMMR	MSI-H	MSI-H
22	40%	15.6	4.8	pMMR	MSS	MSS
23	60%	5.5	4,9	pMMR	MSS	MSS
24	70%	17.1	3.5	pMMR	MSS	MSI-H
25	30%	29.7	6.3	pMMR	MSS	MSS
26	90%	20.5	4.4	pMMR	MSS	MSS*
27	40%	47.1	5.6	pMMR	MSS	MSS
28	85%	18.6	5.5	pMMR	MSS	MSS
29	50%	16.3	4.2	pMMR	MSS	MSS
30	90%	18.2	4.5	pMMR	MSS	MSS
31	90%	4.9	4.5	pMMR	MSS	MSS
32	10%	9.1	3.0	pMMR	MSS	MSS
33	60%	14.2	4.5	pMMR	MSS	MSS
34	60%	13.1	4.1	pMMR	MSS	MSS
35	85%	67.5	5.0	pMMR	MSS	MSS
36	70%	28.1	4.3	pMMR	MSS	MSS
37	80%	69.4	4.9	pMMR	MSS	MSS*
38	65%	6.0	2.8	pMMR	MSS	MSS
39	35%	23.6	3.2	pMMR	MSS	MSS
40	60%	13.5	2.1	pMMR	MSS	MSS
41	40%	14.9	3.3	pMMR	MSS	MSS
42	80%	20.3	2.6	dMMR	MSI-H	MSS
43	80%	13.3	2.4	pMMR	MSS	MSS
44	70%	36.2	3.3	pMMR	MSS	MSS
45	60%	34.4	3.5	pMMR	MSS	MSS
46	70%	24.3	2.5	pMMR	MSS	MSS
47	80%	38.4	2.8	dMMR	MSI-H	MSS
48	40%	65.7	4.3	pMMR	MSS	MSS
49	80%	4.2	1.6	pMMR	MSS	MSS
50	90%	11.8	2.1	pMMR	MSS	MSS
51	90%	62.2	4.8	dMMR	MSI-H	MSS
52	80%	62.0	3.8	pMMR	MSS	MSS
53	70%	42.8	3.4	pMMR	MSS	MSS
54	20%	18.9	3.5	dMMR	MSS	MSS
55	50%	3.4	2.1	pMMR	MSS	MSS

56	75%	11.8	4.3	pMMR	MSS	MSS
57	40%	12.8	3.4	dMMR	MSS	MSS
58	40%	2.6	3.2	dMMR	MSS	MSS
59	70%	10.2	1.3	dMMR	MSS	MSS*
60	75%	5.7	2.2	pMMR	MSS	MSS
61	80%	8.6	2.0	pMMR	MSS	MSS
62	65%	6.4	2.3	pMMR	MSS	MSS
63	60%	9.5	1.8	pMMR	MSS	MSS
64	40%	6.6	2.7	pMMR	MSS	MSS
65	65%	18.5	2.0	pMMR	MSS	MSS
66	70%	52.7	2.3	pMMR	MSS	MSS
67	75%	66.5	2.0	dMMR	MSI-H	MSS*
68	70%	54.5	2.4	pMMR	MSS	MSS
69	85%	16.5	2.2	pMMR	MSS	MSS
70	75%	24.3	2.3	dMMR	MSI-H	MSS*
71	50%	73.5	2.2	pMMR	MSS	MSS
72	80%	24.3	1.7	dMMR	MSI-H	MSS
73	50%	2.69	NE	dMMR	MSI-H	MSS*

Note: *MSI-L. Abbreviations: CRC: colo-rectal cancer; DIN: DNA integrity number; dMMR: deficient mismatch repair; ID: identification number; IHC: immunohistochemistry; MSI-H: high microsatellite instability; MSI-L: low microsatellite instability MSS: microsatellite stable; NA: not assessed; pMMR: proficient mismatch repair.

Supplementary Table S4. Data obtained by comparing results between IHC and TapeStation 4200, IHC and Idylla™, and Idylla™ and TapeStation 4200, taking into account the percentage of acellular mucin.

	**≤50 acellular mucin IHC vs. TapeStation**		
**TapeStation 4200**	**IHC**		**Total**
	**dMMR**	**pMMR**	
**MSI-H**	3 (9.1)	1 (3)	4 (12.1)
**MSS**	7 (21.2)	22 (66.7)	29 (87.9)
**Total**	10 (30.3)	23 (69.7)	33 (100.0)
	kappa=0.31; 95% C.I.: -0.03 to 0.64		
	**>50 acellular mucin IHC vs. TapeStation**		
**TapeStation 4200**	**IHC**		**Total**
	**dMMR**	**pMMR**	
**MSI-H**	7 (17.5)	1 (2.5)	8 (20.0
**MSS**	5 (12.5)	27 (67.5)	32 (80.0)
**Total**	12 (30.0)	28 (70.0)	40 (100.0)
	kappa=0.61; 95% C.I.: 0.33 to 0.88; *p*-value for difference between kappa: 0.182		
	**≤50 acellular mucin IHC vs. Idylla™**		
**Idylla™**	**IHC**		**Total**
	**dMMR**	**pMMR**	
**MSI-H**	8 (24.2)	0 (0.0)	8 (24.2)
**MSS**	2 (6.1)	23 (69.7)	25 (75.8)
**Total**	10 (30.3)	23 (69.7)	33 (100.0)
	kappa=0.85; 95% C.I.: 0.65 to 1		
	**>50 acellular mucin IHC vs. Idylla™**		
**Idylla™**	**IHC**		**Total**
	**dMMR**	**pMMR**	
**MSI-H**	10 (25.0)	1 (2.5)	11 (27.5)
**MSS**	2 (5.0)	27 (67.5)	29 (72.5)
**Total**	12 (30.0)	28 (70.0)	40 (100.0)
	kappa=0.82; 95% C.I.: 0.62 to 1; *p*-value for difference between kappa: 0.836		
	**≤50 acellular mucin Idylla™ vs. TapeStation 4200**		
**TapeStation 4200**	**Idylla™**		**Total**
	**MSI-H**	**MSS**	
**MSI-H**	3 (9.1)	1 (3.0)	4 (12.1)
**MSS**	5 (15.2)	24 (72.7)	29 (87.9)
**Total**	8 (24.2)	25 (75.8)	33 (100.0)
	kappa=0.4; 95% C.I.: 0.03 to 0.78		
	**>50 acellular mucin Idylla™ vs. TapeStation 4200**		
**TapeStation 4200**	**Idylla™**		**Total**
	**MSI-H**	**MSS**	
**MSI-H**	8 (20.0)	0 (0.0)	8 (20.0)
**MSS**	3 (7.5)	29 (72.5)	32 (80.0)
**Total**	11 (27.5)	29 (72.5)	40 (100.0)
	kappa=0.79; 95% C.I.: 0.58 to 1; *p*-value for difference between kappa: 0.072		

Abbreviations: dMMR: deficient mismatch repair; IHC: immunohistochemistry; MSI-H: high microsatellite instability; pMMR: proficient mismatch repair.

Supplementary Table S5. Data obtained by comparing results between IHC and TapeStation 4200, IHC and Idylla™, and Idylla™ and TapeStation 4200, taking into account the stratification by years.

2007-2017 IHC vs. TapeStation 4200

TapeStation 4200	IHC		Total
	dMMR	pMMR	
MSI-H	4 (7.5)	0 (0.0)	4 (7.5)
MSS	11 (20.8)	38 (71.7)	49 (92.5)
Total	15 (28.3)	38 (71.7)	53 (100.0)

kappa=0.34; 95% C.I.: 0.08 to 0.6

2018-2019 IHC vs. TapeStation 4200

TapeStation 4200	IHC		Total
	dMMR	pMMR	
MSI-H	6 (30.0)	2 (10.0)	8 (40.0)
MSS	1 (5.0)	11 (55.0)	12 (60.0
Total	7 (35.0)	13 (65.0)	20 (100.0)

kappa=0.68; 95% C.I.: 0.35 to 1; *p*-value for difference between kappa: 0.136

2007-2017 IHC vs. Idylla™

Idylla™	IHC		Total
	dMMR	pMMR	
MSI-H	11 (20.8)	0 (0.0)	11 (20.8)
MSS	4 (7.5)	38 (71.7)	42 (79.2)
Total	15 (28.3)	38 (71.7)	53 (100.0)

kappa=0.8; 95% C.I.: 0.61 to 0.98

2018-2019 IHC vs. Idylla™

Idylla™	IHC		Total
	dMMR	pMMR	
MSI-H	7 (35.0)	1 (5.0)	8 (40.0)
MSS	0 (0.0)	12 (60.0)	12 (60.0)
Total	7 (35.0)	13 (65.0)	20 (100.0)

kappa=0.89; 95% C.I.: 0.69 to 1; *p*-value for difference between kappa: 0.537

2007-2017 Idylla™ vs. TapeStation 4200

TapeStation 4200	Idylla™		Total
	MSI-H	MSS	
MSI-H	4 (7.5)	0 (0.0)	4 (7.5)
MSS	7 (13.2)	42 (79.2)	49 (92.5)
Total	11 (20.8)	42 (79.2)	53 (100.0)

kappa=0.48; 95% C.I.: 0.17 to 0.78

2018-2019 Idylla™ vs. TapeStation 4200

TapeStation 4200	Idylla™		Total
	MSI-H	MSS	
MSI-H	7 (35.0)	1 (5.0)	8 (40.0)
MSS	1 (5.0)	11 (55.0)	12 (60.0)
Total	8 (40.0)	12 (60.0)	20 (100.0)

kappa=0.79; 95% C.I.: 0.52 to 1; *p*-value for difference between kappa: 0.161

Abbreviations: dMMR: deficient mismatch repair; IHC: immunohistochemistry; MSI-H: high microsatellite instability; pMMR: proficient mismatch repair.

Supplementary Table S6. Data obtained by comparing results between IHC and TapeStation 4200, IHC and Idylla™, and Idylla™ and TapeStation 4200, taking into account DNA concentration.

<25 ng/µl IHC vs. TapeStation 4200			
**TapeStation 4200**	**IHC**		**Total**
	**dMMR**	**pMMR**	
**MSI-H**	10 (18.2)	1 (1.8)	11 (20.0)
**MSS**	9 (16.4)	35 (63.6)	44 (80.0)
**Total**	19 (34.5)	36 (65.5)	55 (100.0)
kappa=0.55; 95% C.I.: 0.32 to 0.79			
**≥25 ng/µL IHC vs. TapeStation 4200**			
**TapeStation 4200**	**IHC**		**Total**
	**dMMR**	**pMMR**	
**MSI-H**	0 (0.0)	1 (5.6)	1 (5.6)
**MSS**	3 (16.7)	14 (77.8)	17 (94.4)
**Total**	3 (16.7)	15 (83.3)	18 (100.0)
kappa=-0.09; 95% C.I.: -0.23 to 0.05; *p*-value for difference between kappa: 0.054			
**<25 ng/µL IHC vs. Idylla™**			
**Idylla™**	**IHC**		**Total**
	**dMMR**	**pMMR**	
**MSI-H**	15 (27.3)	0 (0.0)	15 (27.3)
**MSS**	4 (7.3)	36 (65.5)	40 (72.7)
**Total**	19 (34.5)	36 (65.5)	55 (100.0)
kappa=0.83; 95% C.I.: 0.67 to 0.99			
**≥25 ng/µL IHC vs. Idylla™**			
**Idylla™**	**IHC**		**Total**
	**dMMR**	**pMMR**	
**MSI-H**	3 (16.7)	1 (5.6)	4 (22.2)
**MSS**	0 (0.0)	14 (77.8)	14 (77.8)
**Total**	3 (16.7)	15 (83.3)	18 (100.0)
kappa=0.82; 95% C.I.: 0.49 to 1; *p*-value for difference between kappa: 0.969			
**<25 ng/µL Idylla™ vs. TapeStation 4200**			
**TapeStation 4200**	**Idylla™**		**Total**
	**MSI-H**	**MSS**	
**MSI-H**	10 (18.2)	1 (1.8)	11 (20.0)
**MSS**	5 (9.1)	39 (70.9)	44 (80.0)
**Total**	15 (27.3)	40 (72.7)	55 (100.0)
kappa=0.7; 95% C.I.: 0.48 to 0.92			
**≥25 ng/µL Idylla™ vs. TapeStation 4200**			
**TapeStation 4200**	**Idylla™**		**Total**
	**MSI-H**	**MSS**	
**MSI-H**	1 (5.6)	0 (0.0)	1 (5.6)
**MSS**	3 (16.7)	14 (77.8)	17 (94.4)
**Total**	4 (22.2)	14 (77.8)	18 (100.0)
kappa=0.34; 95% C.I.: -0.17 to 0.85; *p*-value for difference between kappa: 0.172			

Abbreviations: dMMR: deficient mismatch repair; IHC: immunohistochemistry; MSI-H: high microsatellite instability; pMMR: proficient mismatch repair.
